# Prevalence of anti-malarial resistance genes in Dakar, Senegal from 2013 to 2014

**DOI:** 10.1186/s12936-016-1379-2

**Published:** 2016-07-07

**Authors:** Agathe Boussaroque, Bécaye Fall, Marylin Madamet, Khalifa Ababacar Wade, Mansour Fall, Aminata Nakoulima, Khadidiatou Ba Fall, Pierre Dionne, Nicolas Benoit, Bakary Diatta, Yaya Diemé, Boubacar Wade, Bruno Pradines

**Affiliations:** Unité de Parasitologie et d’Entomologie, Département des Maladies Infectieuses, Institut de Recherche Biomédicale des Armées, Brétigny Sur Orge, France; Laboratoire d’étude de la chimiosensibilité du paludisme, Fédération des Laboratoires, Hôpital Principal de Dakar, Dakar, Senegal; Equipe Résidente de Recherche en Infectiologie Tropicale, Institut de Recherche Biomédicale des Armées, Hôpital d’Instruction des Armées, Marseille, France; Unité de Recherche sur les Maladies Infectieuses et Tropicales Emergentes, UM 63, CNRS 7278, IRD 198, Inserm 1095, Aix Marseille Université, Marseille, France; Centre National de Référence du Paludisme, Marseille, France; Service des Urgences, Hôpital Principal de Dakar, Dakar, Senegal; Service de Réanimation Médicale, Hôpital Principal de Dakar, Dakar, Senegal; Service de Pédiatrie, Hôpital Principal de Dakar, Dakar, Senegal; Service de Pathologie Infectieuse, Hôpital Principal de Dakar, Dakar, Senegal; Maternité Hôpital Principal de Dakar, Dakar, Senegal; Chefferie, Hôpital Principal de Dakar, Dakar, Senegal

**Keywords:** Malaria, *Plasmodium falciparum*, Anti-malarial, In vitro, Resistance, Senegal, Molecular marker

## Abstract

**Background:**

To determine the impact of the introduction of artemisinin-based combination therapy (ACT) on parasite susceptibility, a molecular surveillance for antimalarial drug resistance was conducted on local isolates from the Hôpital Principal de Dakar between November 2013 and January 2014 and between August 2014 and December 2014.

**Methods:**

The prevalence of genetic polymorphisms in antimalarial resistance genes (*pfcrt*, *pfmdr1*, *pfdhfr* and *pfdhps*) was evaluated in 103 isolates.

**Results:**

The chloroquine-resistant haplotypes CVIET and CVMET were identified in 31.4 and 3.9 % of the isolates, respectively. The frequency of the *pfcrt* K76T mutation was increased from 29.3 % in 2013–2014 to 43.2 % in 2014. The *pfmdr1* N86Y and Y184F mutations were identified in 6.1 and 53.5 % of the isolates, respectively. The *pfdhfr* triple mutant (S108N, N51I and C59R) was detected in the majority of the isolates (82.3 %). The prevalence of quadruple mutants (*pfdhfr* S108N, N51I, C59R and *pfdhps* A437G) was 40.4 %. One isolate (1.1 %) harboured the *pfdhps* mutations A437G and K540E and the *pfdhfr* mutations S108N, N51I and C59R.

**Conclusions:**

Despite a decline in the prevalence of chloroquine resistance due to the official withdrawal of the drug and to the introduction of ACT, the spread of resistance to chloroquine has continued. Furthermore, susceptibility to amodiaquine may be decreased as a result of cross-resistance. The frequency of the *pfmdr1* mutation N86Y declined while the Y184F mutation increased in prevalence, suggesting that selective pressure is acting on *pfmdr1*, leading to a high prevalence of mutations in these isolates and the lack of specific mutations. The 50.5 % prevalence of the *pfmdr1* polymorphisms N86Y and Y184F suggests a decrease in lumefantrine susceptibility. Based on these results, intensive surveillance of ACT partner drugs must be conducted regularly in Senegal.

## Background

Due to increasing chloroquine resistance, the first-line malaria treatment in Senegal was switched to sulfadoxine–pyrimethamine with amodiaquine in 2004. In 2006, the Senegalese National Malaria Control Programme recommended artemisinin-based combination therapy (ACT) as the first-line treatment for uncomplicated malaria. Therefore, the first-line therapy for uncomplicated malaria became artemether–lumefantrine or artesunate–amodiaquine. The dihydroartemisinin–piperaquine combination was then recommended as a second-line treatment for uncomplicated *Plasmodium falciparum* malaria in Senegal.

Intermittent preventive treatment (IPT) consists of administering sulfadoxine–pyrimethamine and one dose of artesunate during the transmission season and resulted in a 90 % reduction in incidence of clinical malaria in Senegal [1]. Administered once a month to all children or pregnant women, this seasonal IPT can provide a high degree of protection against malaria.

The combination of sulfadoxine–pyrimethamine and amodiaquine was more effective than sulfadoxine–pyrimethamine and artesunate or amodiaquine and artesunate in malaria preventive treatment [2]. During IPT with sulfadoxine–pyrimethamine and piperaquine, only 3.4 % of the treated children had malaria [3].

Since the introduction of ACT and IPT trials in Senegal, very few studies have examined *P. falciparum* resistance to antimalarial drugs. To determine the impact of the introduction of new anti-malarial drugs on parasite susceptibility, a molecular study of anti-malarial drug resistance was conducted on local isolates from the Hôpital Principal de Dakar between November 2013 and January 2014 and between August 2014 and December 2014. The prevalence of genetic polymorphisms in anti-malarial resistance genes, such as the *P. falciparum* chloroquine resistance transporter (*pfcrt*) for chloroquine [4], *P. falciparum* multidrug resistance 1 (*pfmdr1*), which is involved in mefloquine resistance [5] and potentially in quinoline resistance [6, 7], *P. falciparum* dihydrofolate reductase (*pfdhfr*) for pyrimethamine [8] and *P. falciparum* dihydropteroate synthase (*pfdhps*) for sulfadoxine, were evaluated [9].

## Methods

### *Plasmodium falciparum* isolates

In total, 103 symptomatic patients were recruited at the Hôpital Principal de Dakar. Fifty-nine *P. falciparum* isolates were collected between November 2013 and January 2014 and 44 between August 2014 and December 2014. The majority of patients (64 %) were recruited from the emergency department. The other patients were recruited from the intensive care unit (12 %), paediatric department (7 %), infectious diseases department (5 %), maternity department (3 %), and other units (9 %). Anti-malarial treatment prior to admission was not recorded. Despite the WHO’s recommendations, the *P. falciparum* treatment administered at the Hôpital Principal de Dakar until November 2014 was quinine followed by artesunate or artemether–lumefantrine. All the patients or their parents/guardians provided their verbal consent before blood collection. The ethical committee of the Hôpital Principal de Dakar approved the study.

Peripheral venous blood samples were collected in Vacutainer^®^ ACD tubes (Becton–Dickinson, Rutherford, NJ, USA) prior to patient treatment. The diagnosis was performed on thin blood smears stained using a RAL^®^ kit (Réactifs RAL, Paris, France) to determine *P. falciparum* density and to confirm species-specific monoinfection. The level of parasitaemia ranged from 0.001 to 3.3 % in 2013–2014 and 0.06 to 14.1 % in 2014.

### Nucleic acid extraction

Total genomic DNA was extracted from blood sample using a QIAamp DNA Blood Mini Kit according to the manufacturer’s recommendations (Qiagen, Germany).

### Anti-malarial resistance gene single-nucleotide polymorphisms (SNPs)

Four genes, *pfcrt*, *pfmdr1*, *pfdhfr* and *pfdhps*, were amplified by PCR using the reaction conditions described in Table 1 [10–12]. The reaction mixture included 2.5 µL of genomic DNA, 1X reaction buffer (Eurogentec), 200 µM of deoxynucleoside triphosphate mixture (dGTP, dATP, dTTP and dCTP) (Euromedex, Souffelweyersheim, France), variable concentration of MgCl_2_ (Table 1), 0.32 µM of forward and reverse primers and one unit of Red Diamond Taq^®^ DNA polymerase (Eurogentec) in a final volume of 25 µL. The thermal cycler (T3 Biometra, Archamps, France) was programmed as follows: an initial denaturation at 94 °C for 5 min followed by 40 cycles of 94 °C for 30 s, specific hybridization temperature for variable elongation times (Table 1) and 72 °C for extension at 1 min per 1000 bp, and a final 5 min extension step at 72 °C. Purified genomic DNA from *P. falciparum* clone 3D7 was used as a positive control, and water and human DNA were used as negative controls. The reaction products were sequenced using the BigDye Terminator v3.1 Cycle Sequencing Kit (Applied Biosystems) with the primers described in Table 1. The sequencing reaction products were purified using the BigDye XTerminator^®^ Purification Kit (Applied Biosystems) in accordance with the manufacturer’s instructions. Sanger sequencing of PCR products was performed using an ABI Prism 3100 analyser (Applied Biosystems). The sequence data were analysed using Vector NTI Advance™ software (version 11, Invitrogen, Cergy Pontoise, France).

**Table 1:** PCR for anti-malarial resistance genes

Gene	Primers	Amplicon (pb)	[MgCl_2_] (mM)	Tm (°C)	Elongation time (s)
*pfcrt*	Forward CRT76-F	182	4.5	55	20
	TTG GTA AAT GTG CTC ATG TG T				
	Reverse CRT76-R				
	ACA AAT AAA GTT GTG AGT TTC GGA TG				
*pfmdr1*-*1*	Forward MDR1-1F	610	2.5	52	30
	AGA GAA AAA AGA TGG TAA CCT CAG				
	Reverse MDR1-1R				
	ACC ACA AAC ATA AAT TAA CGG				
*pfmdr1*-*2*	Forward MDR1-2F	890	2.5	56	60
	CAG GAA GCA TTT TAT AAT ATG CAT				
	Reverse MDR1-2R				
	CGT TTA ACA TCT TCC AAT GTT GCA				
*pfdhfr*	Forward Dhfr-F	562	2.5	52	20
	ACG TTT TCG ATA TTT ATG C				
	Reverse Dhfr-R				
	TCA CAT TCA TAT GTA CTA TTT ATT C				
*pfdhps*	Forward Dhps-F	971	2.5	53	20
	TTT TGT TGA ACC TAA ACG TG				
	Reverse Dhps-R				
	AAA CGT CAT GAA CTC TTA TTA GAT				

## Results

Of 103 *P. falciparum* isolates collected at the Hôpital Principal de Dakar, 59 isolates were obtained between November 2013 and January 2014 and 44 between August 2014 and December 2014.

The *pfcrt* gene was successfully sequenced in 102 samples. The frequency of the haplotype CVIET was 31.4 % (n = 32 isolates), the haplotype CVMET was 3.9 % (n = 4 isolates) and the haplotype CVMNK was 64.7 % (n = 66 isolates). A molecular resistance profile was identified in 35.3 % of cases (n = 36 isolates), including 29.3 % (n = 17 isolates) in 2013–2014 and 43.2 % (n = 19 isolates) in 2014. The difference is not significant (P value = 0.146, Pearson’s Chi squared test). The CVEMT haplotype was first identified in the 2014 malaria season.

The results for *pfmdr1* polymorphisms are shown in Table 2. The frequency of the 86Y mutation was 5.1 % (n = 5 isolates), and one mixed sample (1 %) harboured both N86 and 86Y alleles. The 184F mutation frequency was 53.5 % (n = 53 isolates), and five of the six isolates harboured both the 86Y and 184F codons. Parasites with the N86 allele and the 184F mutation represented 50.5 % of the isolates. No new SNPs were detected in the *pfmdr1* gene.

**Table 2:** Frequency (%) and number (no) of the *pfmdr1* mutations

Codon	No	Wild type % (no)	Mutated % (no)	Wild type/mutated % (no)
N86Y	99	93.9 (93)	5.1 (5)	1 (1.0)
Y184F	99	46.5 (46)	53.5 (53)	0 (0)
S1034C	102	100 (102)	0 (0)	0 (0)
N1042D	102	100 (102)	0 (0)	0 (0)

The results for *pfdhfr* polymorphisms are presented in Table 3. The mutation frequencies were 87.9 % for the S108N mutation and 85.9 % for both the N51I and C59R polymorphisms. The triple mutant (S108N, N51I and C59R) was detected in 82.3 % of samples.

**Table 3:** Frequency (%) and number (no) of the *pfdhfr* mutations

Codon	No	Wild type % (no)	Mutated % (no)
S108N	99	12.1 (12)	87.9 (87)
N51I	99	14.1 (14)	85.9 (85)
C59R	99	14.1 (14)	85.9 (85)
I164L	99	100 (99)	0 (0)

The results for the *pfdhps* polymorphisms are presented in Table 4. The isolates harboured A437G in 47.2 % of the cases, S436A in 20.2 % of the cases, A613S in 3.2 % of the cases, K540E in 2.1 % of the cases and A581G in 1.1 % of the cases.

**Table 4:** Frequency (%) and number (no) of the *pfdhps* mutations

Codon	No	Wild type % (no)	Mutated % (no)
S436A	89	79.8 (71)	20.2 (18)
A437G	89	52.8 (47)	47.2 (42)
K540E	94	14.1 (97.9)	2.1 (2)
A581G	94	98.9 (93)	1.1 (1)
A613S	94	96.8 (91)	3.2 (3)

The prevalence of the quadruple mutant (*pfdhfr* 108N, 51I, 59R and *pfdhps* 437G) was 40.4 %. One isolate (1.1 %) simultaneously harboured the two *pfdhps* mutations 437G and 540E and the three *pfdhfr* mutations 108N, 51I and 59R.

## Discussion

In total, isolates from only 103 malaria patients were collected in 2013 and 2014 at the Hôpital Principal de Dakar, 55 during the 2013–2014 malaria season and 44 during the 2014–2015 season. This is due to the decreased prevalence of malaria in Senegal (reduction of 27.6 % from 2013 to 2014) [13]. Chloroquine resistance is principally mediated by *pfcrt* mutations in different parts of the world [14]. In this study, the *pfcrt* gene was mutated in 35.3 % of the patients recruited at the Hôpital Principal de Dakar in 2013 and 2014, including 29.3 % of the cases in the 2013–2014 season and 43.2 % of the cases in the 2014–2015 season. This increase in chloroquine resistance has been observed in recent years following a decrease due to the withdrawal of chloroquine and the introduction of ACT in 2002 in Senegal (Table 5). Before the introduction of ACT in Senegal, the prevalence of isolates harbouring the *pfcrt* K76T and in vitro chloroquine resistance was above 50 and 40 %, respectively, in Dakar and its suburb Pikine and in south areas (Dielmo and Ndiop) [15–21]. During the beginning of the ACT implementation (2004–2009), the prevalence of K76T mutant parasites maintained around 50 % [19, 22]. From 2009 to 2011, the prevalence of K76T mutant parasites and in vitro chloroquine resistance decreased to 40 and 25 %, respectively in Dakar [23–25]. Since 2013, the level of chloroquine resistance has increased again to that of 2002 in Dakar [26, 27].

**Table 5:** Molecular (*pfcrt* K76T) and in vitro studies on evaluation of *P. falciparum* susceptibility to chloroquine in Senegal

Year of collection	Site of collection	In vitro chloroquine resistance (%)	*pfcrt* 76T	References
1996	Dielmo/Ndiop	49		[15]
1997–1998	Dielmo/Ndiop	43.5		[16]
1999	Dielmo/Ndiop	55		[17]
2000	Pikine	31	79 %	[18]
2000–2003	Pikine		72.4 %	[19]
2001	Pikine		64 %	[20]
2002	Dakar	52	54 %	[21]
2004–2005	Pikine		47.2 %	[19]
2006–2009	Pikine		59.5 %	[19]
2008–2011	Thies		>50 %	[22]
2009–2010	Dakar	22		[23]
2009–2010	Dakar		37.2	[24]
2010–2011	Dakar	24.2	43.6 %	[25]
2013–2014	Dakar	50		[26]
2013–2014	Dakar		29.3	Present data
2014	Dakar	52.8		[27]
2014	Dakar		43.2 %	Present data

While chloroquine is no longer used in Senegal, the prevalence of in vitro chloroquine resistance and of the *pfcrt* K76T mutation has increased. Two hypotheses could explain the observed increase: (i) the use of artesunate–amodiaquine in Senegal led to the emergence of resistant parasites to amodiaquine; (ii) the development of cross-resistance to chloroquine and monodesethylamodiaquine increased the resistance [23, 28]. This study describes the first detection of the CVMET haplotype in Senegal. In Senegal, the 76T mutation has previously been associated with the CVIET haplotype.

The isolates harbouring the *pfmdr1* mutations 86Y, 184F, 1034C and 1042D were identified in 6.1, 53.5, 0 and 0 % of the patients, respectively. The prevalence of 86Y has decreased over the past few years in Senegal from >30 % in 2000 to 6 % in 2013–2014 (Table 6) [18–20, 22, 24, 25]. Since 2010, the prevalence of parasites harbouring the 184F mutation has remained stable and above 50 % in Dakar [24, 25]. This prevalence has more than doubled from 30 % in 2008 to greater than 70 % in 2011 in Thiès [22]. The frequency of the *pfmdr1* mutation N86Y declined, while the frequency of the Y184F mutation increased, suggesting that selective pressure is acting on *pfmdr1*, leading to a high prevalence in these isolates and the lack of specific mutations. The role of polymorphisms in *pfmdr1* is still debated. The 86Y mutation was associated with increased in vitro susceptibility of *P. falciparum* parasites to dihydroartemisinin, lumefantrine, monodesethylamodiaquine and mefloquine [29]. In contrast, this *pfmdr1* 86Y mutation was associated with a decrease of in vitro susceptibility to dihydroartemisinin, lumefantrine, and mefloquine in *P. falciparum* isolates from Asia [30], Kenya [31] and Benin [32]. Field studies in east Africa have also shown selection of the N86 allele in recurrent infections after treatment with artemether plus lumefantrine [33–36] or artesunate plus mefloquine [37], which suggests that N86 is a marker of in vivo lumefantrine resistance. In addition, parasites harbouring both the N86 and 184F alleles were less susceptible to lumefantrine and mefloquine in vitro [29]. This profile was detected in 50.5 % of the isolates collected in Dakar.

**Table 6:** Evolution of *pfmdr*1 N86Y mutation in *P. falciparum* parasites in Senegal

Year of collection	Site of collection	N86Y	References
2000	Pikine	31 %	[18]
2001	Pikine	30.6 %	[20]
2002–2003	Pikine	40 % (about)	[19]
2005–2009	Pikine	20 % (about)	[19]
2009	Thiès	20 % (about)	[22]
2009–2010	Dakar	17.2 %	[24]
2010–2011	Dakar	16.1	[25]
2011	Thiès	<5 %	[22]
2013–2014	Dakar	6.1 %	Present data

Conversely, the *pfmdr1* 86Y mutation has been shown to be associated with in vivo amodiaquine resistance during recrudescence after amodiaquine monotherapy [38] or after combination therapy with artesunate–amodiaquine [39]. The odds ratio for amodiaquine therapeutic failure associated with the 86Y mutation was 5.4 [40]. These results may suggest a low prevalence of *P. falciparum* strains resistant to amodiaquine in Dakar. These data are in contrast with previous studies in Senegal: (i) the 86Y mutation was significantly associated with increased susceptibility to monodesethylamodiaquine, the active metabolite of amodiaquine, in parasites collected from 2009 to 2011 in Dakar [29]; (ii) the prevalence of isolates with reduced in vitro susceptibility to monodesethylamodiaquine increased significantly from 5.6 % in 2013 to 30.6 % in 2014 with an increase in IC_50_ values from 9.8 to 25.3 nM [26, 27]; (iii) the re-increase of *P. falciparum* strains resistant to chloroquine appears to be due to cross-resistance between in vitro susceptibility to chloroquine and monodesethylamodiaquine [23, 28] and the use of artesunate–amodiaquine in Senegal, which can generate the emergence of parasites resistant to amodiaquine. The increase in amodiaquine IC_50_ values was already observed in isolates collected in Thiès between 2008 and 2011 [22].

The *pfdhfr* 108N mutation has been found to be correlated with in vitro and in vivo resistance to pyrimethamine [5, 41]. The risk of therapeutic failure with sulfadoxine–pyrimethamine was greater for patients harbouring the 108N mutation (odds ratio of 3.5) during a 28 day follow-up [40]. Additional mutations—51I (odds ratio of 1.7) or 59R (odds ratio of 1.9)—increase the level of in vitro resistance to anti-folate drugs and sulfadoxine–pyrimethamine [40]. The risk of in vivo resistance to sulfadoxine–pyrimethamine increased by 4.3 with the triple mutation (108N, 51I and 59R) [40]. In 2013–2014, the prevalence of the *pfdhfr* 108N mutation was 87.9 % in malaria patients who were treated at the Hôpital Principal de Dakar. *dhfr* triple mutants at codons 51I, 59R and 108N were associated with high-level pyrimethamine resistance and represented 82.3 % of the isolates. Since 2002, the prevalence of triple mutants has increased from 50 to 82.3 % in 2013–2014 (Table 7). This increase was observed in different areas of Senegal [3, 42, 43].

**Table 7:** Evolution of *pfdhfr* 51I, 59R and 108N triple mutation in *P. falciparum* parasites in Senegal

Year of collection	Site of collection	51I, 59R, 108N (%)	References
2002	Dakar	50	[21]
2003	Pikine	61	[42]
2003	Thies	40	[43]
2007	Keur Soce	67	[3]
2009–2010	Dakar	75.3	[24]
2010–2011	Dakar	73.6	[25]
2011	Thiès	93	[43]
2013–2014	Dakar	82.3	Present data

The *pfdhps* 437G mutation has been shown to be correlated with in vitro and in vivo resistance to sulfadoxine [6]. The risk of therapeutic failure with sulfadoxine–pyrimethamine increased by 1.5 and 3.9 with the single mutation A437G and the double mutation A437G and K540E, respectively [40]. In 2013–2014, 47.2 % of the isolates harboured the 437G mutation in Dakar. This prevalence increased after 2002 and was then stable from 2009 to 2014 with 40–50 % of the isolates harbouring the 437G mutation in Senegal (Table 8). Several studies from 2006 to 2008 in Senegal showed that the prevalence of *pfdhps* 437G significantly increased after IPT of infants with sulfadoxine–pyrimethamine [3, 44]. Only two isolates (2.1 %) carried the double mutation (437G and 540E) that is associated with high-level sulfadoxine resistance. The *pfdhps* mutation of codon 613 (A613S) (3.2 %) is very rare in Africa.

**Table 8:** Evolution of *pfdhps* A437G mutation in *P. falciparum* parasites in Senegal

Year of collection	Site of collection	A437G (%)	References
2002	Dakar	20	[21]
2003	Pikine	40	[42]
2009–2010	Dakar	40.4	[24]
2010–2011	Dakar	47.5	[25]
2013–2014	Dakar	47.2	Present data

In Dakar, the prevalence of isolates harbouring the quadruple mutants (*dhfr* 108N, 51I, 59R and *dhps* 437G) was stable from 2009 to 2014: 36.5 % in 2009, 36.7 % in 2010 and 40.4 % in 2014 [24, 25]. In Thiès, the prevalence of quadruple mutants increased from 20 to 66 % between 2003 and 2011 and then dropped to 44 % in 2013 [43]. In 2010, the quadruple mutants were identified in 79.4 % of the isolates from areas of Senegal where sulfadoxine–pyrimethamine plus amodiaquine were administered to children during seasonal malaria chemoprevention versus 67.1 % in areas where they were not treated [45]. In 2014, only one isolate harboured the *pfdhps* mutations 437G and 540E and the *pfdhfr* mutations 108N, 51I and 59R; these mutations are associated with high-level sulfadoxine–pyrimethamine resistance. These findings suggest that regular surveillance of molecular markers should be performed in areas where IPT with sulfadoxine–pyrimethamine is used.

In summary, the prevalence of chloroquine resistance continues to increase after a decline due to the official withdrawal of the drug and the introduction of ACT. Furthermore, amodiaquine susceptibility may be decreased as a result of cross-resistance. The frequency of the *pfmdr1* mutation N86Y declined while the frequency of the Y184F mutation increased, suggesting that selective pressure is acting on *pfmdr1*, leading to a high prevalence in these isolates and the lack of specific mutations. The 50.5 % prevalence of the *pfmdr1* polymorphisms N86 and 184F suggests a decrease in lumefantrine susceptibility. Based on these results, intensive surveillance of ACT partner drugs must be conducted regularly in Senegal.

Furthermore, molecular surveillance in Dakar has demonstrated the emergence of polymorphisms in the K13 propeller domain gene, which is associated with in vitro and in vivo resistance to artemisinin in Asia [46–48]. However, these mutations detected in Dakar and more generally in Africa have not yet been associated with artemisinin resistance.

## Abbreviations

ACT: artemisinin-based combination therapy
*pfcrt*: *Plasmodium falciparum* chloroquine resistance transporter gene
*pfmdr1*: *Plasmodium falciparum* multidrug resistance 1 gene
*pfdhfr*: *Plasmodium falciparum* dihydrofolate reductase gene
*pfdhps*: *Plasmodium falciparum* dihydropteroate synthase
IPT: intermittent preventive treatment
WHO: World Health Organization
SNPs: single-nucleotide polymorphisms
DNA: deoxyribonucleic acid
